# Empowering roots—Some current aspects of root bioenergetics

**DOI:** 10.3389/fpls.2022.853309

**Published:** 2022-08-16

**Authors:** Lars H. Wegner

**Affiliations:** International Research Center for Environmental Membrane Biology, Foshan University, Foshan, China

**Keywords:** bioenergetics, mitochondria, alternative oxidase, biochemical pH clamp, root architecture, aerenchyma, salinity

## Abstract

Roots of higher plants provide the shoot with nutrients and water. In exchange, they receive photosynthates, which serve both as energy source and building blocks for maintenance and growth. While studies in plant bioenergetics used to focus on photosynthesis, several more recent findings also aroused or renewed interest in energy conversion and allocation in roots. Root building costs were identified as a long-undervalued trait, which turned out to be highly relevant for stress tolerance and nutrient use efficiency. Reduced building costs per root length (e.g., by aerenchyma formation or by increasing the cell size) are beneficial for exploring the soil for nutrient-rich patches, especially in low-input agrosystems. Also, an apparent mismatch was frequently found between the root energy budget in the form of the ATP pool on the one side and the apparent costs on the other side, particularly the costs of membrane transport under stress conditions, e.g., the Na^+^ detoxification costs resulting from Na^+^ sequestration at the plasma membrane. Ion transport across the plasma membrane (and also endomembranes) is coupled to the proton motive force usually believed to be exclusively generated by H^+^ ATPases. Recently, an alternative mechanism, the biochemical pH clamp, was identified which relies on H^+^ formation and binding in the apoplast and the cytosol, respectively, driven by metabolism (so-called active buffering). On this background, several aspects of root bioenergetics are discussed. These are (1) root respiration in soil, with a critical view on calorimetric vs. gas exchange measurements; (2) processes of energy conversion in mitochondria with a special focus on the role of the alternative oxidases, which allow adjusting carbon flow through metabolic pathways to membrane transport processes; and (3) energy allocation, in particular to transport across the plasma membrane forming the interface to soil solution. A concluding remark is dedicated to modeling root bioenergetics for optimizing further breeding strategies. Apparent “energy spoilers” may bestow the plant with a yet unidentified advantage only unfolding their beneficial effect under certain environmental conditions.

## Introduction

Life persists by a constant flow of free energy—when this energy flow and its tightly regulated partitioning comes to an end, this is, in fact, a hallmark of death. So, life ceases as soon as the process of energy conversion is irreversibly disrupted. By contrast, biological *matter* and structures remain intact and in place for a short while, varying from a few seconds up to days, before degradation sets in. Consistently, Dupré and Nicholson (2018), proponents of a process philosophy in the life sciences, agued “that the living world is a hierarchy of processes stabilized and actively maintained at different timescales,” rather than one of “things” (i.e., matter). For a less drastic variation of this topic, we may refer to a situation of perfect homeostasis of a cell or an organism implying a living, but invariant state, (which should be considered a thought experiment and will hardly be encountered in reality). Life is operating far from equilibrium, and maintaining homeostasis is necessarily associated with constant energy dissipation and, often inseparably connected, also a flow of matter. A compelling case study was recently communicated by Dreyer (2021): Using a simple modeling approach, he showed that K^+^ homeostasis in individual plant cells is necessarily associated with a free energy-consuming circular flow of K^+^ across the plasma membrane. These considerations imply that it is bioenergetics, which is concerned with the very key processes of life, namely studying and analyzing the life-preserving energy flow and energy conversion. This is, in fact, the most concise definition of bioenergetics—for more details see Demirel and Sandler (2002) and Nicholls (2013). The significance of energy conversion is most obvious for plants which have (with few exceptions) monopolized the conversion of solar photon radiation into chemical energy, making the primary energy source on earth available to all forms of life. Therefore, plant bioenergetics is often exclusively associated with processes directly related to photosynthesis, while subsequent processes of energy conversion in the plant body such as respiration (oxidative phosphorylation) receive much less interest and tend to be undervalued. In this short review, the focus will be on the bioenergetics of roots.

Raising the issue of root bioenergetics provokes the initial inquiry “what is a root?” Not uncommon in the biological sciences, basic morphological/functional categories such as “root” become the fuzzier the closer you look at them, due to the diversity of life forms and the evolutionary radiation of morphological and anatomical adaptations in the plant kingdom (Graham et al., 2000). Luckily, we can refer to an excellent treatise of this issue by Raven and Edwards, who raised the same question some 20 years ago (Raven and Edwards, 2001). The most straightforward answer we can give to this question is related to function: Roots (i) anchor a plant in soil, providing mechanical stability (used in an allegorical sense in common language), (ii) provide an interface for exchange with soil, allowing the plant to extract water and nutrients, subsequently translocated to the shoot by long-distance transport *via* xylem vessels. As pointed out by Raven and Edwards (2001), these functions can also partly or fully by taken over, e.g., by rhizomes (if it comes to stability) or mycorrhiza (with respect to nutrient acquisition; Salvioli di Fossalunga and Novero, 2019). On the other hand, roots are not necessarily only below-ground organs. In contrast to shoots, their extension growth is strictly apical and in all vascular plants the meristematic tissue is protected by a root cap. Segmentation by nodes is never observed, in contrast to shoots (Raven and Edwards, 2001).

For our purpose, we can leave aside “exceptions” (even though it can be argued what is considered as “exceptional”; Groff and Kaplan, 1988) and address higher plants as typically possessing above-and below-ground organs which operate in a more or less strict “division of labor”: Large parts of the aboveground parts perform photosynthesis making use of solar radiation and transforming this energy into energy-rich organic chemical bounds. Below-ground organs are excluded from this energy source and rely on a transfer of photo-assimilates from the shoot, usually in the form of sucrose transported *via* the phloem. In exchange, below-ground organs scavenge water and nutrients from the soil (in cooperation with mycorrhiza and microorganisms). Below-ground organs with this function will in the following be addressed as “roots” irrespective of exact morphological categories.

In the framework of this division of labor, roots, like all non-photosynthetic plant organs, are generally considered as an “investment” of the plant relying on constant feeding with products of photosynthesis, predominantly in the form of sucrose, both as building blocks, and as a source of energy. Revenues are expected in the form of new sources of water and nutrients made accessible by root growth, leading to a continuous expansion of the root system. According to a common heuristic principle widely used in (plant) ecophysiology for more than 4 decades (Bloom et al., 1985), life is thought to follow economic paradigms expressed by an adequate nomenclature. Lynch (Lynch and Ho, 2005; Lynch, 2007a) even introduced a new research field he coined “rhizoeconomics.” The analogy is grounded on all life forms being continuously exposed to a selection pressure, “forcing” them to allocate limited resources in an economical way, and to reduce costs. “Benefits” and “trade-offs” are interpreted with respect to reproductive success and viability of the individual or the species. Species can follow different “strategies” for optimizing these parameters. Although this line of thinking has proven very successful in the past, it also tends to convey an anthropomorphic and teleonomic bias (see also the last section of this review).

**Figure 1:**
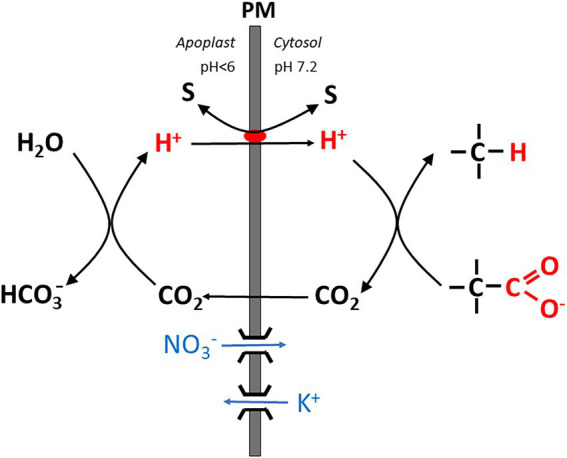
Simplified scheme showing the basic mechanism of the “biochemical pH clamp” in a root cell. H^+^ is generated in the apoplast when bicarbonate is formed from CO_2_. Upon translocation into the cytosol, H^+^ is resorbed by a biochemical reaction which involves decarboxylation of an organic acid. CO_2_ is released by this reaction and passes through the membrane into the apoplast. Thus, a pH gradient is maintained across the membrane by active buffering, which can be harnessed to drive a solute “S” across the membrane, either in symport or antiport with H^+^. Charge balance is maintained by nitrate influx or K^+^ efflux *via* ion channels. PM, plasma membrane.

Interest in the bioenergetics of plants, and particularly of root systems, has been revived recently on several grounds two of which will be treated here in more depth:

1. A new quest for energy-and nutrient-efficient crops has directed attention to hitherto undervalued anatomical features of the root. The search opted for more robust crops allowing to minimize the use of fertilizers, which is important both from an ecological and an economical viewpoint. This work was pioneered by JP Lynch and coworkers (Lynch, 1995, 2007b, 2013). They highlighted the metabolic costs of soil exploration for water and nutrients and identified them as key traits for a resource-efficient agriculture, which is of particular importance in developing countries (Lynch, 2015). In particular, building costs per root length, a parameter that had been largely ignored hitherto, were addressed. A large cell volume was found to be beneficial (Colombi et al., 2019) since it tends to minimize, e.g., cell wall material required per root volume, whereas mechanical stability of root tissue is hardly compromised because it predominantly relies on the turgor pressure which is largely independent of cell size. Interestingly, formation of aerenchyma, e.g., in maize, which was hitherto only discussed in the context of oxygen supply to root tissue under waterlogging conditions (Wegner, 2010), also reduces metabolic costs of root expansion considerably (Lynch, 2015; Lynch et al., 2021) without compromising root mechanical properties too much unless soil compaction imposes special requirements on root stability (Colombi et al., 2019). Cost reduction is also obtained by root cortical senescence leading to a complete degradation of the root cortex in some Poaceae, or as a consequence of secondary growth in dicots (Lynch et al., 2021). It could be shown that this effect is indeed harnessed under natural conditions to minimize metabolic costs of soil exploration for nutrient-rich plaques—this had been identified as conveying considerable evolutionary benefits, since a large share of the daily assimilated C is invested into root growth. In the slow-growing grass *Festuca ovina*, e.g., it is about 40%. Some 25% of photosynthates is allocated to structural material used for root growth, and another 15% is spent on root growth-related respiration (including ion uptake; calculation based on data of Atkinson and Farrar, 1983 and Lambers et al., 2002).
  In a study on the bioenergetics of salinity, Munns et al. (2020a) calculated the energetical benefit associated with reducing the thickness of the root cortex from two cell layers (as, e.g., in wheat branch roots) to a single layer (corresponding to the situation in *Arabidopsis* roots) when roots were exposed to salinity. They found a 20% lower ATP consumption due to the reduction in plasma membrane surface which, in turn, led to a reduction in the costs of Na^+^ exclusion per root surface. A reduction in membrane surface reduces Na^+^ influx and, hence, costs of Na^+^ detoxifications. A re-organization of root morphology under saline conditions, favoring the development of branch roots while seminal root growth was hampered, is in accordance with this rationale. Arsova et al. (2020) observed a 70% lower energy requirement for branch roots per unit root length in wheat compared to seminal roots. These energy savings were associated with a severe reduction of transpiration-driven water uptake during daytime, though. This reminds us of the fact that energy savings usually come with a cost due to adverse side effects (see also the last section, further below). Energetic implications of root architecture and development will certainly receive continued interest in the near future, with a focus on modeling approaches like those of Munns et al. (2020a) and Arsova et al. (2020). When discussing root morphology and its adaptation to nutrient demand we should also be aware that most plant species (four out of five) rely on symbiosis with mycorrhiza for efficient nutrient foraging (Salvioli di Fossalunga and Novero, 2019), and root branching, apical growth, and root/shoot ratio are strongly affected by fungal colonization (Hughes et al., 2008). Among the three common types, i.e., arbuscular, ericoid, and ectomycorrhiza, the first is the most widespread and best studied one. Particularly the hyphae of arbuscular mycorrhiza operates as an extension of the root system, allowing to mine the soil for nutrients with low mobility, particularly P and K, but also facilitating the uptake of N. In return, the fungus is provided with monosaccharides and fatty acids by its host, thus providing building blocks and a source of energy. Specific building costs for fast hyphal growth are much lower compared to fine roots which are about 10 times thicker and possess a complex multicellular anatomy (Fitter, 1991). Hence, extending the root system with these symbionts is likely to be beneficial for the plant from an energetic point of view, even though for a cost-and-benefit evaluation it has to be taken into account that long-distance transport in hyphae is less effective than in the xylem conduits of roots.
2. Particularly under stress conditions such as salinity, an apparent mismatch between energy demand (being higher than in the absence of these stresses), and the root energy budget in terms of ATP production by oxidative phosphorylation was frequently observed. An instructive case study was provided by Foster and Miklavcic (2020) modeling costs of nutrient and water transport as well as Na^+^ detoxification under saline conditions in *Arabidopsis* roots. In their model calculations based on the available experimental data, the apparent energy demand for membrane transport at the plasma membrane of cortical and stelar cells, and the root tonoplast exceeded the available ATP pool by a factor of at least 2. By varying the abundance of membrane transporters, costs could be lowered to somewhat more realistic values for the cortical plasma membrane only, whereas no cost reduction was attained for the other membranes. Experimental data obtained for circular Na^+^ transport under saline conditions had also previously provided evidence for a high energy demand exceeding the free energy available in the form of the root ATP pool (Malagoli et al., 2008). This brought some authors to the conclusion that data on circular Na^+^ transport across the plasma membrane of root cortical cells (obtained with radioactive tracers) are fallacious and represent ion exchange processes in the apoplast instead (e.g., Munns et al., 2020b). Foster and Miklavcic (2020) also stated that “since our model is based on currently understood and accepted ion transport mechanisms, our predictions suggest there is a need to re-assess these mechanisms.” When ATP production does not keep up with energy dissipation by membrane transport processes, transport could be fueled instead by processes which do not involve proton pump activity, i.e., do not rely on ATP hydrolysis. Na^+^ secretion from cortical cells back into the root apoplast operates against a more or less steep Na^+^ concentration gradient and is fueled by antiport with H^+^ *via* SOS1 or another type of antiporter, taking advantage of the pH gradient across the membrane which favors H^+^ influx. Recently Wegner and Shabala (2020) suggested that this antiport could be energized by an alternative mechanism denoted as the “biochemical pH clamp” (Figure 1). By a process called active buffering, a stable pH gradient across the membrane (with apoplastic and cytosolic pH values around 5.5 and 7.2, respectively) could be maintained despite continuous H^+^ influx required, e.g., for Na^+^ export from the cytosol even if this influx is not balanced by pump-driven H^+^ extrusion. The biochemical pH clamp involves continuous H^+^ release in the apoplast and H^+^ scavenging in the cytosol *via* biochemical reactions. In the apoplast, H^+^ is formed by conversion of CO_2_ (previously released by oxidative phosphorylation) to HCO_3_^−^. H^+^ then serves as a substrate for SOS1 (or another type of antiporter) and is channeled into the cytosol by exchange with Na^+^. In the cytosol, H^+^ is subsequently bound again by a biochemical reaction involving a decarboxylation reaction, which is the case with those catalyzed by malic enzyme and glutamate decarboxylase. A more detailed account will be given in the section. “The energetics of membrane transport” further below. Note that in contrast to the limited buffer capacity of apoplast and cytosolic matrix, active buffering is, in principle, inexhaustible because substrates are continuously replenished by metabolism.

In the following, recent work related to root bioenergetics will be discussed in light of more than 60 years of research on this issue. The journey will start with whole-plant physiology, and with ecophysiological work on roots in their natural environment (i.e., in soil), including respiration measurements. Subsequently, energy metabolism will be treated with a focus on more recent work on mitochondria and oxidative phosphorylation. Then, the bioenergetics of plasma membrane transport will be treated in some depth. Finally a prospect on possible directions of future research will be given.

## Observations on the whole plant level

As a starting point for a more comprehensive treatise of root bioenergetics, we may choose energy export from source tissues in the shoot, mainly leaves, to the root. Photosynthetic products, predominantly sucrose, are shuttled to the root *via* mass flow in the phloem. Simple equations can be used to estimate the related transfer of free energy (which has to be attained by photosynthesis). From the phloem flow velocity, the virtual cross sectional area of the phloem and sucrose concentration in the xylem sap, the rate of sucrose transfer from shoot to root, is accessible. From these data, the free energy transfer rate can be estimated by inserting the energy that can potentially be harvested from one sucrose molecule by energy metabolism.


(1) $$J_{suc}=v_{\mathrm{ph}}*A_{Ph}*c_{suc,Ph}$$

(2) $$J_{E}=J_{suc}*G_{suc}$$

With $J_{suc}$ = sucrose flux from shoot to root; $v_{Ph}$ = phloem flow velocity; $A_{Ph}$ = conductive phloem cross section at the shoot base; $c_{suc,Ph}$ = sucrose concentration in phloem sap; $J_{E}$ = free energy flux from root to shoot; $G_{suc}$ = free energy of sucrose. The most exact and reliable data on phloem flow velocity can be obtained by MRI imaging allowing non-invasive flow monitoring. For *Ricinus communis*, Peuke et al. (2006) obtained a flow velocity of ~0.3 mm s^−1^. Similar values for flow velocity ranging from 0.25 to 0.4 mm s^−1^ were reported for *Ricinus* and three other species [poplar, tomato, and tobacco; Windt et al. (2006)]. Volume flow was in the range of 0.1 mm^3^ s^−1^ for tomato and tobacco and 0.2 mm^3^ s^−1^ for *Ricinus*; only in poplar, values were somewhat higher (around 0.9 mm^3^ s^−1^). At a sucrose concentration of phloem sap around 0.44 M in *Ricinus* (Peuke et al., 2006), we obtain a sucrose transport velocity of ~80 nmol s^−1^ for this species. A Gibbs free energy of 5,795 kJ/mol for sucrose renders an energy transport rate of 0.46 J s^−1^. The above approach provides us with something like an upper limit of the true energy transfer rate from shoot to root, since sucrose is not fully oxidized to CO_2_. It has to be kept in mind that sugars also play an important role as building blocks/precursors, e.g., for amino acids, lipids, and ribonucleotides (see also further below). Even if the material required for growth can be quantified, residual sucrose is not fully available for energy metabolism due to root exudation of organic acids (Wegner et al., 2021), and the rapid turnover of enzymes and other molecules. A fraction of 15–67% of total carbohydrates allocated to the root is invested into energy metabolism, i.e., root respiration (Table 1 in Lambers et al., 2002).

A more promising approach to quantify root energy metabolism is provided by directly monitoring root respiration which is usually quantified by measuring CO_2_ release and/or O_2_ consumption. Both parameters can be assessed by gas exchange measurements in soil. They are related by the respiratory quotient (RQ), which is supposed to be close to 1 when sucrose is the exclusive substrate of energy metabolism (Lambers and Oliveira, 2019). RQ can exceed this value, e.g., when malate synthesized in the shoot is transported to the root and decarboxylated there (see also further below), and with nitrate assimilation occurring in the root. The last two decades have seen some technical advance in this field, including chamber techniques for measuring soil respiration with more efficient CO_2_ detectors (Rochette and Hutchinson, 2005). Numerous studies have been published focusing on measuring respiratory activity in soil samples, mostly by assessing CO_2_ release. The contribution of roots to soil respiration was shown to vary considerably, ranging from 8 to 64% (Wang and Fang, 2009). Separating root respiration from other biological activity, microbial and fungal respiration, is a major challenge in ecological research (Ruehr and Buchmann, 2010). Root respiration can be “subtracted” from total soil respiration by making use of root exclusion chambers surrounded by meshes of μm pore size which cannot be invaded by root growth. Additionally, the contribution of fine roots can be estimated by separately determining respiration on root samples and calculating their overall contribution on the basis of fine root biomass. In a mixed forest close to Zurich (Switzerland) with beech, ash, fir, spruce, lime, maple, oak, and elm trees, these measurements revealed a strong dependence of root respiration on plant phenology and photosynthetic activity (Ruehr and Buchmann, 2010). Temperature dependence of root respiration in tree stands reported previously may at least partly depend on such phenological effects. In recent years, this technique has been refined to assess mycorrhizal CO_2_ release separately from the contribution of roots (both usually being summarized as autotrophic respiration) and from heterotrophic bacterial activity by using meshes of a pore size of ~50 μm which allow penetration of hyphae and exclude roots, in addition to fully closed and fully accessible root collars. Mycorrhizal CO_2_ emission was shown to be relevant for overall soil respiration, but its share varied strongly. It depended, among other things, on the type of mycorrhiza and the ecosystem that was investigated. For example, in a larch forest (*Larix kaempferi*) about 6% of total soil respiration was ascribed to mycorrhiza (roots 42%; Makita et al., 2021) and in a barley field it was 4.8% [roots 25%; Moyano et al. (2007)]. Larger shares were found, e.g., for an apple orchard (mycorrhiza 11%, roots 12%; Tomè et al., 2016) in a lodgepole pine forest (mycorrhiza 25%, roots 15%; Heinemeyer et al., 2007), and in a stand of Norway spruce (mycorrhiza 18–44%, roots did not contribute significant under most conditions; Neumann and Matzner, 2014). In the latter two studies, ectomycorrhiza were dominant. An average value of 15% mycorrhizal contribution to total soil CO_2_ emission was recently reported in a metastudy by Han et al. (2021). A detailed record on all aspects of soil respiration, including its relevance for global change modeling approaches, can be obtained from Luo and Zhou (2006). Soil emission is the largest CO_2_ source on earth, and its management is considered highly relevant for limiting global temperature increase.

Recently, calorimetric techniques have been advocated as an alternative approach to monitoring CO_2_ release for a broader and more precise quantitative assessment of root energy metabolism (Colombi et al., 2019; Herrmann and Colombi, 2019). Model calculations generally rely on the assumption of CO_2_ release being driven by oxidative phosphorylation, implying that O_2_ supply is not a limiting factor. However, soil O_2_ partial pressures can be highly inhomogeneous and, hence, root tissue may locally be in a hypoxic state, undetected by bulk measurements. Indeed, heat dissipation can be measured with a high precision and comparatively little effort and provides an interesting alternative to gas exchange measurements for assessing root respiration. However, the underlying concept needs to be considered with some care. Calculations based on calorimetric measurements rely on the assumption of zero net energy input in a biological system residing in a “steady state” (which *de facto* comes down to the absence of growth). Free energy input *via* the phloem under these conditions is hypothesized to be eventually fully transduced to heat. This is definitely an oversimplification, since sugars are still needed as building blocks, e.g., for the synthesis of root exudates which are secreted into the soil (Wegner et al., 2021). Moreover, free energy required for maintaining steep concentration gradients with the environment remains unconsidered (see the Introduction). This energy does not contribute to heat release, but rather counteracts the entropy-driven free energy loss by (electro)diffusion (Wegner, 2015). According to the theoretical framework used by proponents of the calorimetric method, in a growing tissue catabolic (=dissipative) processes leading to heat production are complemented by an anabolic component which is equal to the energy stored in biomass surplus, as, e.g., determined by combustion. For relating heat dissipation to CO_2_ release (and, in turn, to glucose consumption) a calorespirometric ratio is defined; under aerobic conditions a value of 30 kJ per g CO_2_-C is assumed, but under hypoxic conditions this value is thought to double. Even though these values are based on empirical measurements, it is at least questionable if they can be treated as constants. Heat dissipation by metabolism will depend, e.g., on the role of the alternative oxidase in respiratory processes in mitochondria (see further below). Moreover, the local differences in oxygen supply in soil emphasized by the proponents of the calorimetric method are hard to quantify and hamper the interpretation of calorimetric data in terms of glucose consumption.

Despite methodological difficulties and limitations, a combination of field ecological studies and whole plant physiology lab experiments has considerably extended our understanding of autotrophic soil respiration in recent decades (Hopkins et al., 2013). Generally, the rate of CO_2_ release from roots follows photo-assimilation in aboveground organs, as expected for a source limitation and blockage of assimilate transport by stem girdling in trees impairs it. Consistently, leaf pruning, e.g., in wheat (Bingham and Stevenson, 1993), significantly reduces specific root respiration while in bean, removal of pods increases it (Fanello et al., 2020), as root pruning does in the roots that remain attached (Bingham and Stevenson, 1993; Fanello et al., 2020). This provides evidence for a competition between sinks. Cellular adenylate levels provide for a fine regulation by both regulating glycolysis and the mitochondrial electron transport chain [Lambers et al. (2002); see also the following section]. A large fraction of root maintenance costs is invested into nitrate uptake and assimilation (Bouma and De Visser, 1993; Bouma et al., 1994; Scheurwater et al., 1998; Hopkins et al., 2013), and root N has even been employed as a proxy for fine root respiration in some ecological studies (Jia et al., 2011).

## Processes of energy conversion in mitochondria

**Figure 2:**
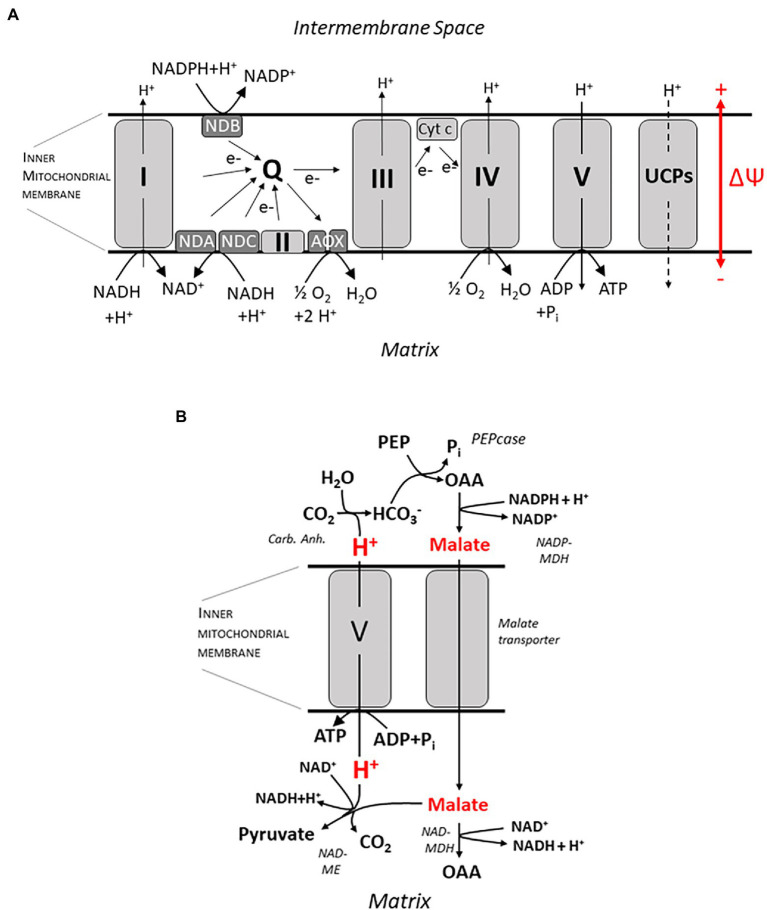
ATP synthesis at the inner mitochondrial membrane. In **(A)** an overview on the electron transport chain is given, including complexes I–V, NAD(P)H dehydrogenases (ND) A, B and C and the alternative oxidases (AOX). Q, ubichinone pool; UCPs, uncoupling proteins; and ΔΨ, membrane potential. A putative mechanism for ATP synthesis which does not involve circular H^+^ flow is shown in **(B)**. H^+^ is released by formation of HCO_3_-catalyzed by the (Carb. Anh.) reaction. HCO_3_-is subsequently assimilated by the Phosphoenolpyruvate-Carboxylase (PEPcase) synthesizing Oxaloacetate (OAA) from Phosphoenolpyruvate (PEP). Subsequently malate is formed by the NADPH-dependent Malate-Dehydrogenase (NADP-MDH), and malate passes through a yet unidentified transporter into the mitochondrial matrix. Subsequently, it can either be oxidized again to oxaloacetate by the NAD-dependent Malate-Dehydrogenase in the matrix (NAD-MDH), or it is decarboxylated to pyruvate by the NAD-dependent Malic Enzyme (NAD-ME) while binding a H^+^. Thus, a pH gradient is built up across the membrane which is harnessed by F0F1 ATPase (complex V) to form ATP. For more details, see text.

Ecophysiological work on root bioenergetics, highlighting, among other things, the relevance of anatomical and morphological traits for the bioenergetics of nutrient acquisition, has renewed attention to this field, which received only marginal interest by plant physiologists for quite a while. For a quantitative approach, a profound knowledge of energy metabolism down to the molecular scale is certainly required to understand its fine-tuning in response to a plethora of individual physiological conditions. This includes nutrient availability, photosynthetic activity in source tissues, and challenges by biotic and abiotic stressors, which may act in various intensities and combinations. Indeed, tremendous progress has also been made in this field, particularly with respect to research on plant mitochondria which play a key role in energy conversion. Basic principles of the chemiosmotic theory for ATP synthesis relying on a proton motive force (pmf) across the inner mitochondrial membrane date back to the 60s of the last century, and the organization of the electron transport chain with four redox complexes, three of which (I, III, and IV) translocating H^+^ from the mitochondrial matrix into the intermembrane space, and with an intermediate ubichinone pool was unraveled in the 70 and 80s (Figure 2A). This basic knowledge will not be covered here, and the reader is referred to excellent description in textbooks (e.g., Nicholls, 2013; Lambers and Oliveira, 2019). In more recent work, the focus shifted to the genetic basis and molecular structure of these complexes and, most prominently, to their regulation. Interest in the latter was stimulated by the growing awareness for challenges to the stability of energy transduction processes in mitochondria, e.g., by the generation of reactive oxygen species (ROS) when the proton motive force and/or the membrane potential at the inner mitochondrial membrane shift into a critical range (Wagner and Moore, 1997). Research into the regulation of electron transport in mitochondria was initiated some 50 years ago when an alternative oxidase (AOX) was identified shown to be insensitive to the inhibitor cyanide and transferring electrons from the ubichinone pool to O_2_ (Figure 2A) without contributing to the trans-membrane pH gradient (Bendall and Bonner, 1971). The small family of AOX proteins consists of two subfamilies, AOX1 and AOX2, both of which are encoded by nuclear genes. Monocots usually only possess AOX1 and lack the AOX2 subfamily. AOX proteins include a four-helix bundle coordinating binuclear di-iron center which is able to bind and activate oxygen (Moore et al., 2013; May et al., 2017). Current evidence indicates that AOX exists as a homodimer. Reduction of disulfide bounds induce a transition from an inactive state to a “dormant” state of low activity readily mobilized by other factors like intermediates of the tricarboxylic acid (TCA) cycle (Vanlerberghe et al., 2020). The inner mitochondrial membrane also contains (alternative) NAD(P)H dehydrogenases (NDs), which are insensitive to the inhibitor rotenone. Alternative NDs accept electrons from NAD(P)H and feed them into the ubichinone pool in the same way as the complex I (the “ordinary” NAD(P)H dehydrogenase; Figure 2A), but without translocating H^+^. Two dehydrogenases, NDA and NDC, are associated with the inner surface of the mitochondrial membrane, whereas NDB is located at the outer surface (Sweetman et al., 2019). Acting sequentially, NDs and AOXs transfer electrons from NAD(P)H to O_2_ without converting the energy into a chemiosmotic gradient; rather it is dissipated as heat. Thus the “standard” electron transport chain is completely short-circuited, as demonstrated for *Arabidopsis* (Clifton et al., 2005, 2006). A bypass also exists for the F0F1 ATP synthase: The uncoupling proteins (UCPs) found, e.g., in potato tubers channel H^+^ and dissipate the pmf, but do not contribute to ATP synthesis (Hourton-Cabassa et al., 2004). The role of these enzymes apparently just “spoiling” the redox equivalents provided by metabolism has puzzled researchers for decades. Numerous studies devoted to unraveling their function (usually focusing on the AOX) have been published since then (Moore and Rich, 1980; Wagner and Krab, 1995; Millenaar and Lambers, 2003; Vanlerberghe, 2013; Vanlerberghe et al., 2020). From these publications, we can distill several non-exclusive explanations: (i) Experimentally well founded is a role of the alternative oxidase for heat generation, e.g., in spade leaves of the Araceae, which employ volatile compounds to attract insects for pollination (Wagner et al. 2008). However, this specialized function does not explain the general presence of these enzymes in plant mitochondria, including root cells. Some authors speculated on a more widespread function of AOX in long-term temperature adjustment, though [summarized in Vanlerberghe (2013)]; (ii) It has been pointed out that part of the energy metabolism (and certainly glycolysis) has (at least) a dual function: Transforming the free energy stored in C-H and C-O bonds into a “universal currency,” namely ATP (catabolic function), *and* providing building blocks for the synthesis of biomolecules such as peptides, fatty acids, and DNA [anabolic function; (Millenaar and Lambers, 2003; Vanlerberghe, 2013)]. The latter requires a sufficient throughput of enzymatic pathways, particularly the glycolysis and part of the TCA cycle, which, in turn, relies on a fast recovery of NAD(P)^+^, the oxidized form of the nicotinamides. This is the function of the electron transport chain in the inner mitochondrial membrane. Its potential blockage by low rates of ATP synthesis, e.g., due to low phosphate availability or low energy demand, may have a far-reaching systemic effect and feedback on the rates of biomolecule synthesis. According to this explanation, alternative oxidases have the function to guarantee a minimum NAD(P)H throughput in the mitochondria to keep the anabolic metabolism going, thus having a role in both redox balancing and carbon skeleton production. Vanlerberghe (2013) has re-evaluated this point, stating that AOX has a role in fine-tuning growth with nutrient efficiencies, particularly with respect to P_i_. Phosphate deficiency tends to limit ATP synthesis. This could entail an energization of the inner mitochondrial membrane favoring the generation of reactive oxygen species (ROS) which threaten protein integrity (Wagner and Moore, 1997). Hence, a third major function of the AOX has been advocated, namely (iii) serving as a security valve for controlling the pH gradient and membrane potential across the mitochondrial inner membrane (both contributing to pmf) as well as the redox state (MP) of the electron transport chain, among other things for limiting ROS production. A large voltage drop of 220–250 mV (cytosol relative to matrix) was obtained by probing the MP, e.g., for mitochondria isolated from potato tubers, whereas the ΔpH was relatively small [0.2 units; Ducet et al. (1983)], and the membrane potential of individual mitochondria is controlled to minimize ROS production (Schwarzländer et al., 2012). Consistently, it was found that a low matrix pH activates AOX (Millenaar and Lambers, 2003), and external ROS have been shown to stimulate expression of AOX1. Usually matrix pH is at about 7.8.

The current state of discussion on the AOXs already highlights that there is no single answer to the question for its physiological function. However, some important findings are still left unconsidered or did not meet a satisfactory explanation yet. The most striking still unexplained finding is related to the AOX regulation under stress conditions. For conditions of drought stress (Costa et al., 2007) and salinity (Smith et al., 2009; Martí et al., 2011, Mhadhbi et al., 2013, Munns et al., 2020a) a significant *increase* in AOX activity has repeatedly been reported despite the fact that under these conditions additional energy is required to synthesize or accumulate osmotica and to detoxify the cytosol by dumping Na^+^ in the vacuole or the apoplast, respectively. Hence, the efficiency of energy metabolism is expected to increase under stress conditions, apparently precluding any need for a non-functional energy *dissipation*. Focusing on salinity, a solution to this conundrum may come from the recent suggestion of Wegner and Shabala (2020) for an energization of membrane transport by direct coupling to energy metabolism, with no ATP hydrolysis directly involved. According to this “biochemical pH clamp” hypothesis already brought up in the Introduction (Figure 1), transport coupled to H^+^ influx across the plasma membrane (such as antiport with Na^+^) can be driven by biochemical reactions generating and capturing H^+^ in the apoplast and in the cytosol, respectively. It is obvious that these active buffering processes must be tightly coupled to the metabolic carbon flow, and the AOX can adjust this flow according to need, de-coupling it from the actual rate of ATP hydrolysis. It is important to note that the ATP pool is always shared among various functions, the main ones being maintenance, growth, and membrane transport (Poorter et al., 1991; Amthor, 2000). Instead, the biochemical pH clamp exclusively fuels membrane transport, with no competition by the other consumers (Wegner and Shabala, 2020). This exclusiveness makes it particularly valuable for coping with stress conditions.

Previously, the biochemical pH clamp has been discussed as a mechanism for energizing membrane transport across the plasma membrane of root cells (Wegner and Shabala, 2020; Wegner et al., 2021), and this topic will be addressed in more detail in the following section. Here, I would like to suggest an additional role for this mechanism in ATP *synthesis* in mitochondria which has, to my knowledge, not been considered before. ATP synthesis *via* the F0F1 ATP synthase (complex V; Figures 2A,B) requires a sufficient chemiosmotic gradient across the inner mitochondrial membrane which is, according to a generally accepted dogma, exclusively generated by the electron transport chain (e.g., Vanlerberghe, 2013), translocating 3 H^+^ per NADH molecule oxidized. It is an important corollary of this dogma, which I would like to challenge here, that no ATP can be synthesized at the inner mitochondrial membrane when the electron transport chain does not contribute to the pmf—as with the alternative ND and the AOX bypassing the “ordinary” transport chain. Under these conditions, H^+^ transport for ATP synthesis could still be organized as a *linear* flux, though, purportedly organized in the following way: In the cytosol, malate is synthesized from phosphoenolpyruvate by the PEP carboxylase and the malate dehydrogenase, with oxaloacetate serving as an intermediate (Figure 2B). This involves the release of 1 H^+^ per turnover. Malate subsequently enters the mitochondrial matrix by facilitated (electro) diffusion and is subsequently decarboxylated by the NADH-dependent malic enzyme, leading to the formation of pyruvate, at the expense of 1 H^+^. Pyruvate is then fed into the TCA cycle. Note that this reaction sequence gives rise to a pH gradient across the inner mitochondrial membrane, which could be harnessed to drive a H^+^ influx into the mitochondrial matrix and, in turn, ATP synthesis. For the stoichiometry of the F0F1 ATPase, a ratio of 4 H^+^ per ATP has been determined (Steigmiller et al., 2008). The necessary molecular “infrastructure” for this reaction sequence has indeed been verified, and several experimental observations lend support to this scenario. When protocols for the isolation of viable mitochondria, e.g., from cauliflower buds and potato tubers, had been worked out in the 70s of the last century, it was soon observed that providing these mitochondrial preparations with external malate plus NAD^+^ as substrates, respiratory activity was stimulated as monitored by O_2_ consumption, providing evidence that malate readily diffuses into the matrix (Macrae and Moorhouse, 1970). Two enzymes were shown to compete for malate as a substrate (Figure 2B), namely the NADH-dependent malate dehydrogenase (NAD-MDH, directly catalyzing a step in the TCA cycle) and malic enzyme (NAD-ME). Under most conditions, the contribution of the ME was marginalized by the MDH as determined by immediately measuring oxaloacetate vs. pyruvate levels (Rustin et al., 1980). Even when the MDH was blocked, the ME could only partly take over its role (Le et al., 2021a), and only a small fraction of ME-derived pyruvate was channeled into the TCA cycle (Le et al., 2021b). However, at high AOX activities, ME was the dominant enzyme. This even made researchers believe (erroneously) that AOX and the NADH-dependent malic enzyme were tightly associated and part of a separate redox pool (Rustin et al., 1980) but note recent data by Le et al. (2021b) suggesting that pyruvate imported *via* the pyruvate carrier (MPC) complex may not mix with that generated by the (NAD-ME). Interestingly, pyruvate, the product of the ME, directly activates the AOX when the dimer is in the reduced state (Day et al., 1994; Vanlerberghe, 2013). ME is also activated when the final electron acceptor O_2_ becomes less available during hypoxia (Edwards et al., 1998). These experimental findings are in accordance with the hypothesis forwarded here. Another piece of evidence refers to malate flux which is supposed to carry negative charges for electrically balancing the H^+^ transfer, thus avoiding any drift in membrane potential. Indeed, evidence for the existence of an electrogenic malate transporter not identical to or related with the MPC was obtained, even though its genetic basis still appears to be unidentified (Le et al., 2021a). A further critical corollary of the hypothesis is a *net* H^+^ influx into mitochondria in the presence of malate—this should be accessible with the Microelectrode Ion Flux Estimation (MIFE) technique (Shabala et al., 2006).

Special aspects of mitochondrial function discussed above have highlighted the complexity of mitochondrial activity for plant metabolism, even beyond its key role in bioenergetics. Much progress has been made recently in understanding how mitochondria are “programmed” according to the actual status of the organism and its current requirements. An interesting aspect of this programming is the coordination of genetic information stored on the nucleus and on the mitochondrion. In recent years, the role of mitochondria in sensing stress and coordinating the cellular response by activating nuclear genes was gradually recognized, now known as the “mitochondrial retrograde response” (Wagner et al., 2018). A wide spectrum of nuclear genes, including those encoding for AOX1, is activated by inhibiting electron transport with Antimycin A, which acts on complex III and stimulates ROS production. The response to this chemical as well as other specific inhibitors like rotenone and oligomycin has been analyzed in quite detail, including transcription factors of the NAC family which coordinate the response. Interestingly, they originate from the ER and move into the nucleus after release into the cytosol in response to mitochondrial dysfunction. Inhibitors seem to mimic “natural” effects on electron transport by stress such as ozone or drought, and response to hypoxic conditions which also tend to block electron transport. Re-organization of energy metabolism induced by a lack of oxygen is a separate issue and will not be treated in more depth here. The reader is referred to recent review articles by Lee and Bailey-Serres (2021) and Loreti and Perata (2020).

Finally, recent findings on oxidative phosphorylation in arbuscular mycorrhiza will be treated briefly. Invasion of the fungus by endobacteria frequently adds a third partner to the symbiosis (Salvioli et al., 2016). Having a reduced genome and metabolism, these bacteria fully rely on their host, which itself depends on the supply of sugars by the plant upon root inoculation. As a model system, the fungus *Gigaspora margarita* was investigated which is infected by the bacterium Candidatus *Glomeribacter gigasporarum* (CaGg.; Salvioli et al., 2016; Vannini et al., 2016) Interestingly, the endobacterium stimulates, among other things, fungal ATP synthesis by activating enzymes of the inner mitochondrial membrane and activates pathways for ROS scavenging alternative to those operating in fungi cultures lacking endobacteria. The root infection process and antioxidant activities in the root were also affected (Venice et al., 2017).

## The energetics of membrane transport

The previous section centered on the question how energy is provided and made accessible in the roots of higher plants, particularly dealing with processes in mitochondria. Efficient “management” of the ATP pool while, at the same time, minimizing hazards, e.g., by excessive ROS production turned out to be an important trade-off. Alternative mechanisms of energy conversion not involving the ATP pool were also mentioned, but so far rather *en passant*. In the following, the focus shifts to the way metabolic energy is “spent” on and allocated to basic root functions. The plasma membrane of cortical root cells and root hairs will receive most attention here, since they form the interface to soil solution and play a key role in nutrient and water acquisition. Furthermore, they are “in the first line of defense” against biotic and abiotic stresses. As a consequence, steep concentrations gradient for numerous solutes need to be established and maintained across the plasma membrane of these cells, either for accumulating them in the cytoplasm (as in the case of nutrients such as K^+^) or to prevent their uptake, which applies to potentially harmful solutes such as Na^+^ and Cl^−^. In plant cells, membranes are generally energized by establishing a proton motive force across them, consisting of a pH gradient and an electrical membrane potential. For all other solutes, energetically uphill transport is coupled to the pmf, either to the pH gradient (as in the case of SOS1 catalyzing an electroneutral exchange of H^+^ and Na^+^), or to the membrane potential driving, e.g., K^+^ into the cell *via* ion channels even against a concentration gradient, or to both. At the plasma membrane, P-type H^+^ ATPases play a key role in pmf generation by pumping H^+^ against its electrochemical gradient at the expense of splitting an energy-rich phosphate from ATP, ending up with ADP and P_i_ (Briskin et al., 1995; Falhof et al., 2016). The stoichiometry is generally 1 H^+^ per ATP hydrolysis (Briskin and Reynolds-Niesman, 1991), but a value of 3 was obtained under conditions of salinity stress (Janicka-Russak et al., 2013), indicating that the pump can also operate in a more efficient mode. P-type ATPases are encoded by the AHA gene family (for AUTOINHIBITED PLASMA MEMBRANE PROTON ATPases; Falhof et al., 2016; Hoffmann et al., 2019). In root cortical cells, AHA2 is expressed besides the constitutively present AHA1 and appears to be involved in nutrient acquisition. AHA7 is additionally expressed specifically in root hairs. AHA2 activity was maximal at a cytosolic pH of about 6.4 and strongly decreased when the pH was elevated to physiological values close above 7 (Hoffmann et al., 2019); still, it was clearly involved, e.g., in phosphate uptake in *Arabidopsis* roots and contributed to the acidification of the root surface in the elongation zone (Yuan et al., 2017). Similarly, in *Arabidopsis* roots it played a role in Fe acquisition (Santi and Schmidt, 2009). AHA7 differed from AHA2 with respect to its pH dependence: It showed full activity around cytosolic values around 7 (close to the physiological range), but was strongly inhibited at external pH values below ~pH 6.0; this is due to an extracellular autoinhibitory loop.

For a long time, P-type H^+^ ATPases have been considered as the *exclusive* pmf source at the plasma membrane of plant cells, until experiments with the MIFE technique provided compelling evidence that this is not always the case. Under various experimental conditions summarized by Wegner and Shabala (2020), a *net* H^+^ *influx* was measured into root cells for 2 h at least, indicated that H^+^ flux by symporters and antiporters apparently *exceeded* active H^+^ efflux mediated by the proton pump. Net influx was even recorded when fluxes were monitored at opposite sites of the same protoplast, with no cell wall potentially interfering with the measurement. Apparently, a pmf is maintained under these conditions despite the ongoing H^+^ influx (Figure 1). As described in the Introduction, this is explained by active buffering which involves H^+^ release and resorption in the apoplast and in the cytosol, respectively, by metabolism. Active buffering stabilizes the pH gradient during net H^+^ influx (also denoted as the “biochemical pH clamp,” see above). Note that H^+^ influx is also associated with a positive charge transfer from the apoplast into the cytosol. Charge balance can either be achieved by simultaneous ion-channel mediated influx of anions (e.g., nitrate) and/or by K^+^ efflux. The former will remain an exceptional case, and hence continuous K^+^ release is likely to be a corollary of the biochemical pH clamp, unless the overall charge balance for all transport processes is neutral. Hence, the process will come to an end when the vacuolar K^+^ pool is exhausted. This will take several hours (Wu et al., 2018). We have to conclude that the mechanism is not self-sustained, but requires an intermittent refilling of the cellular K^+^ pool, possibly at night.

In the apoplast, a pH drift to values >~6 is prevented by the activity of an acid carbonic anhydrase. One H^+^ is released when bicarbonate is formed from H_2_O and CO_2_, the latter being an end-product of respiration (see above). Moreover, the adjacent soil provides a huge H^+^ reservoir whereas limited cell wall buffering only plays a minor role (the specific buffer capacity amounting to just about 10% of the cytosolic one; Felle (2001)) and can only transiently counteract a pH shift. All these mechanisms of pH control provide just for a rather loose clamp, and apoplastic pH can fluctuate to some extent, also allowing it to act as a second messenger e.g. under drought stress and hypoxia (Felle, 2001; Geilfus, 2017) and for growth regulation (Shao et al., 2021). Moreover, local pH gradients can prevail, e.g. perpendicular to the plasma membrane (Martinière et al., 2013) and between roots zones (Peters and Felle, 1999).

**Figure 3:**
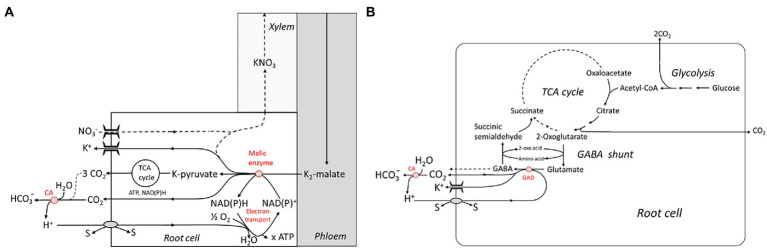
Metabolic schemes showing “active” buffering in the cytosol by malic enzyme and the electron transport chain **(A)** and the glutamate decarboxylase (GAD) which is part of the 4-Aminobutyric acid (GABA) shunt **(B)**. Both systems are capable of capturing H^+^ imported by cotransport with or antiport against other substrates (“s”). Extracellular buffering is brought about by the carbonic anhydrase (CA). TCA, tricarbonic acid. Reproduced from Wegner and Shabala (2020), with permission.

On the other hand, H^+^ *scavenging* in the *cytosol* requires biochemical reactions involving a decarboxylation step. Two rather complex reaction schemes have been advanced by Wegner and Shabala involving the NADPH-dependent ME and the glutamate decarboxylase (GAD), respectively (Figures 3A,B). The first one closely follows the well-known Ben-Zioni Lips model of anion circulation in higher plants (Zioni et al., 1971). According to this model, nitrate taken up by the root is transported to the leaves for N assimilation *via* the transpiration stream. K^+^, serving as a counterion, is subsequently cycled back to the root *via* the phloem. Charge balance in phloem transport is obtained by shoot-to-root transfer of malate, which is decarboxylated in the root, forming pyruvate (Figure 3A). The latter step involves binding of H^+^ which enters the cytosol *via* various cotransporters. Note that malate derived from *glucose* is associated with the release of 4 H^+^ and, hence, subsequent decarboxylation does not result in overall net H^+^ consumption. However, when malate synthesis takes place in the leaves, alkalinization by photosynthetic nitrate assimilation is elegantly neutralized. Another metabolic pathway that is highly stimulated at various stress conditions, e.g., by salinity, and consuming H^+^ is the so-called GABA shunt. GABA, or 4-aminobutyric acid, is a zwitterionic, non-protein amino acid that is found in almost all plants species and tissues. It is produced *via* an anaplerotic pathway branching from the TCA cycle that involves trans-amination of 2-oxoglutarate leading to the formation of glutamate, and subsequent decarboxylation (Figure 3B). The latter reaction is catalyzed by the Glutamate decarboxylase (GAD) and is associated with binding of one H^+^ ion. GABA can be fed back into the TCA cycle by a two-step process yielding succinate. Alternatively, it is released into the apoplast—in fact, GABA has been identified as a signaling molecule for communication between individual plants (Ramesh et al., 2017). The pivotal role of the GABA shunt in preventing acidification of the cytosol is known since the pioneering work of Crawford (Crawford et al., 1994), but these authors did not consider the link with the energization of membrane transport processes. Interestingly, cytosolic acidification stimulates the GAD, and, in turn, GABA formation, e.g., in carrot suspension cells (Carroll et al., 1994; Crawford et al., 1994) originally derived from storage root tissue (Israel and Steward, 1967). For a review, the reader is referred to Shelp et al. (1999). GABA concentrations in the cytosol can be as high as 6–39 mM under stress conditions such as drought stress and salinity (Renault et al., 2010). Strong evidence supporting a role of the H^+^ scavenging GABA shunt in salinity stress tolerance was obtained by Nikolas Taylor and coworkers (Che-Othman et al., 2020). They reported on a complete re-organization of the TCA cycle with NaCl treatment in wheat leaves. Under these conditions, the GABA shunt was the preferred route, avoiding the 2-oxoglutarate dehydrogenase complex (OGDC) that was completely disintegrated when exposed to elevated Na^+^ concentrations. This study was performed on leaf cells, but evidence for a similar mechanism operating in *Arabidopsis* roots had previously been presented (Renault et al., 2013). The observations are in line with a key role of the GAD in capturing H^+^ entering the cytosol in exchange for Na^+^. It should be noted that these biochemical pH stat mechanisms do not exert a tight control on cytosolic pH either; it can vary to some extent, allowing it to act as a second messenger (Felle, 2001), as described above for the apoplastic pH.

Thermodynamic aspects of the biochemical pH clamp hypothesis were discussed in detail by Wegner and Shabala (2020), estimating the metabolic free energy that needs to be invested into stabilizing the trans-membrane H^+^ gradient for counteracting continuous H^+^ influx. Among other things, it was pointed out that the decarboxylation step catalyzed by ME would even be slightly energetically uphill under standard conditions; hence, this reaction could only drive H^+^ influx at a favorable ratio of products to educts. Since ME simultaneous decarboxylates *and* oxidizes malate, the reaction strongly depends on the redox state of the cell controlling the NADP^+^/NADPH ratio. Hence, H^+^ resorption is likely to depend on continuous oxidation of NADPH by the mitochondrial electron transport chain, possibly also involving the AOXs (see the previous section). Decarboxylation of glutamate catalyzed by GAD is slightly exergonic, the free energy balance being ~ − 11 kJ/mol under standard conditions (calculated on the basis of data from Efe et al., 2008), and the whole GABA shunt is also associated with a release of free energy.

In addition to the plasma membrane, endomembranes are another relevant “energy sink,” not only in roots. In the first place, this refers to vacuoles (Davies, 1997) which are used as intracellular storage sites for various solutes. Transport across the tonoplast is energized by V-type ATPases, which share many properties with their counterparts in the plasma membrane albeit with some remarkable exceptions such as their variable stoichiometry, and the Pyrophosphatases being specific for the tonoplast. Interestingly, cycling of Na^+^ (and possibly of other solutes like K^+^) is also likely to play a role at the tonoplast and may cause very high energetic costs at this membrane, too (Shabala et al., 2020).

## The energetics of root growth

Transport and storage are important energy investments in the root, another very important one is growth (for recent reviews see Dupuy et al., 2018, Volkov et al., 2021). Root growth and building costs were already treated in the Introduction to motivate root bioenergetics as a timely issue. Exploring the heterogeneous soil for nutrient-rich sites relies on maintaining root growth at a high rate; this is of particular importance for getting access to resources of low mobility like phosphate (Lynch and Ho, 2005; Lynch, 2015, 2022), whereas mobile nutrients, e.g., N, can be mobilized more readily by the transpiration stream. In this context, building costs of root tissue per root length was identified as a key trait for optimizing the root growth rate. This can be expressed in terms of a carbon economy for soil exploration (Nielsen et al., 2001), which was indeed shown to be affected, e.g., by phosphate availability. Parameters like root diameter, cell size, and aerenchyma formation have been shown to be relevant. On the other hand, a detailed physiological comparison of slow and fast-growing grass species [which is a genetically encoded property, persisting at identical nutrient supply; Poorter et al. (1991)] revealed that metabolic energy spent on root growth normalized to dry weight was almost identical, e.g., in fast-growing *Dactylus glomerata* and slow-growing *F. ovina*. However, *total* respiration did not scale with the growth rate, the fast-growing species being apparently more efficient [2.3 times faster growth at a factor of 1.2 for the specific respiration rate; Scheurwater et al. (1998)]. The difference was rather related to N uptake which consumed less energy in the fast-growing species than in the slow one due to a lower rate of N circulation across the plasma membrane of root cortical cells.

Anatomical and morphological traits leading to reduced specific building costs of roots have been well characterized, but their genetic basis is frequently still elusive, maybe with the exception of aerenchyma formation relying on programmed cell death (Evans, 2004). Only few genes have been identified so far which modify root system architecture in a clearly defined way (for reviews, see Wachsman et al., 2015; Uga, 2021). Among those is the gene DEEPER ROOTING 1 (DRO1) in rice encoding for a protein which has an impact on the angle at which the root growth relative to the gravitational field of the earth (and, hence, on the rooting depth) and PSTOL1, which speeds up lateral growth of rice roots in P-deficient soils. A more detailed evaluation of the genetic basis of root anatomical and morphological adaptation to the energy status as well as nutrient availability is expected in the years to come.

## Final remarks

Numerous “construction sites” were identified in this short review on various aspects of root energetics which follow their own agenda. I do not want to be repetitive returning to these special topics, but raise a more fundamental issue instead. Traits leading to an efficient use of energy have been identified as highly beneficial from an evolutionary point of view, and crop breeding is being re-oriented toward that goal (De Block and Van Lijsebettens, 2011; Lynch, 2015; Munns et al., 2020b). A new twist was added to the field by introducing traits which had been undervalued so far such as an “energy-efficient” root architecture and anatomy (see Introduction). Another “mega-concept” is concerned with aspects of use efficiency of nutrient elements (N, S, and P) which also has bearing on the bioenergetics of plants (Lynch, 2019). Definitely, these approaches are promising and justified—still, I want to add a note of caution. Plants, as life in general, operate far from thermodynamic equilibrium and their existence relies on a constant flow of free energy (see the Introduction). Hence, they are not *per se* designed as “energy savers”—rather, they tend to gain biological function by “spending,” or, rather, “investing” energy, in order to get some value in return with respect to fitness and viability. What at first sight appears as a waste of energy may in effect convey a hidden function. In fact, it may turn out as an important security valve proving its value at certain stress scenarios only. We have to be careful avoiding anthropomorphisms when judging plant “success” in “saving energy.” The ongoing discussion on AOX and the other “energy spoilers” in mitochondrial electron transport (see section “Processes of energy conversion in mitochondria”) provide a telling example. Apparently, plants have developed sophisticated mechanisms for—tightly regulated—energy *dissipation*, which is in obvious contrast to energy *efficiency* being, under all circumstances, a key trait for fitness. Another interesting case study refers to the breeding of salt-tolerant crops. The apparently “futile” cycling of Na^+^ at the plasma membrane of root cortical cells has been identified as an important energy sink, and eliminating this trait has been defined as a goal in breeding salt-tolerant crops (Munns et al., 2020b). However, if this Na^+^ cycling has a function in water acquisition as discussed by Munns et al. (2020a) loosing this trait will imply the loss of a potentially important function, with unforeseen costs and consequences for the entire organism. In fact, ionic cycling across the plasma membrane can be an investment into maintaining cellular homeostasis (Dreyer, 2021; see also the Introduction). We also have to keep in mind that under many environmental conditions, the availability of energy is not the rate-limiting factor for plant survival—rather, it is the availability of water, certain nutrients, or the avoidance of biotic or abiotic stressors. This is particularly true for (crop) plants growing in warm climates. Lynch has stressed the importance of searching for traits which allow for a resource-efficient agriculture, particularly in developing countries (Lynch, 2007b, 2015, 2022), but an efficient use of *energy* may not always be the major concern. At least this may not be a general concern, and hidden trade-offs need to be considered. For example, P deficiency tends to come with an energy deficit due to its effect on the adenylate availability, which will feed back on the respiration rate (see section “Processes of energy conversion in mitochondria”). In a recent broad meta-analysis, Han and Zhu (2021) investigated correlations between root morphological traits and root respiration and did not find a convincing relationship for traits like specific root length and root diameter, suggesting that interactions may be more complex and species-dependent. New approaches in quantitative modeling of plant performance with respect to certain parameters such as energy use efficiency will become available in the near future, such as those pioneered by Foster and Miklavcic (2020) and Arsova et al. (2020). Instead of just opting for “energy-efficient” plants, the task will rather be to find the best trade-off between energy efficiency and function, and to find algorithms reflecting this trade-off in the best way.

## Author contributions

The author confirms being the sole contributor of this work and has approved it for publication.

## Funding

This work was supported by a grant of the National Science Foundation of China to LW (grant no. 32070277).

## Conflict of interest

The author declares that the research was conducted in the absence of any commercial or financial relationships that could be construed as a potential conflict of interest.

## Publisher’s note

All claims expressed in this article are solely those of the authors and do not necessarily represent those of their affiliated organizations, or those of the publisher, the editors and the reviewers. Any product that may be evaluated in this article, or claim that may be made by its manufacturer, is not guaranteed or endorsed by the publisher.
